# Periodontal microorganisms and Alzheimer disease – A causative relationship?

**DOI:** 10.1111/prd.12429

**Published:** 2022-03-04

**Authors:** Gert Jungbauer, Alexandra Stähli, Xilei Zhu, Lavinia Auber Alberi, Anton Sculean, Sigrun Eick

**Affiliations:** ^1^ Department of Periodontology School of Dental Medicine University of Bern Bern Switzerland; ^2^ Private Dental Practice Straubing Germany; ^3^ Swiss Integrative Center for Human Health Fribourg Switzerland

## Abstract

In the initiation or exacerbation of Alzheimer disease, the dissemination of oral microorganisms into the brain tissue or the low‐level systemic inflammation have been speculated to play a role. However, the impact of oral microorganisms, such as *Porphyromonas gingivalis*, on the pathogenesis of Alzheimer disease and the potential causative relationship is still unclear. The present review has critically reviewed the literature by examining the following aspects: (a) the oral microbiome and the immune response in the elderly population, (b) human studies on the association between periodontal and gut microorganisms and Alzheimer disease, (c) animal and in vitro studies on microorganisms and Alzheimer disease, and (d) preventive and therapeutic approaches. Factors contributing to microbial dysbiosis seem to be aging, local inflammation, systemic diseases, wearing of dentures, living in nursing homes and no access to adequate oral hygiene measures. *Porphyromonas gingivalis* was detectable in post‐mortem brain samples. Microbiome analyses of saliva samples or oral biofilms showed a decreased microbial diversity and a different composition in Alzheimer disease compared to cognitively healthy subjects. Many in‐vitro and animal studies underline the potential of *P gingivalis* to induce Alzheimer disease‐related alterations. In animal models, recurring applications of *P gingivalis* or its components increased pro‐inflammatory mediators and β‐amyloid in the brain and deteriorated the animals' cognitive performance. Since periodontitis is the result of a disturbed microbial homoeostasis, an effect of periodontal therapy on the oral microbiome and host response related to cognitive parameters may be suggested and should be elucidated in further clinical trials.

## 1 INTRODUCTION

Alzheimer disease (AD) has drawn considerable critical attention due to its significant impact on human beings and its high costs to society. In 2019, AD and other forms of dementia ranked as the 7th leading cause of death around the world and affected disproportionately more female and elderly patients.^1^ Globally, 65% of deaths from AD and other forms of dementia are women. An estimated 6.2 million Americans aged 65 years and older are suffering from AD in 2021^2^ and this trend is expected to escalate further in the coming decades up to 13.85 million by 2060.^3^ AD is a type of chronic progressive brain disease. It starts with unnoticeable changes in the brain and then gradually develops into symptoms such as memory loss, cognitive dysfunction, and language problems as soon as neurons have been damaged or partly destroyed. As the neuronal damage is progressive and irreversible, impaired communication, disorientation, and behavioral disorder gradually appear. Eventually, patients are unable to speak, swallow, or walk.^3^, ^4^, ^5^

AD is divided into two classes or categories, early onset AD (EOAD) and late onset AD (LOAD) based on a cutoff age at 65 years when disease features start to appear in individuals.^6^, ^7^ EOAD accounts for around 5.5% of all AD cases.^6^, ^8^ It is believed that mutations in the amyloid precursor protein (APP), presenilin 1 (PSEN1), and presenilin 2 (PSEN2) are high‐risk factors for EOAD,^9^, ^10^ but only 10%‐15% of EOAD cases can be explained in that way.^7^, ^11^ Recent research indicates that most EOAD cases are related to a mix of common and rare autosomal recessive variants, such as sortilin‐related receptor (SORL1) and triggering receptor expressed on myeloid cells   (TREM2),^12^ while LOAD tends to be a multicomponent result related to genetic and environmental factors with higher prevalence.^13^ Apolipoprotein ε4 allele (APOE4) is the only major susceptibility gene for LOAD but the genetic architecture of LOAD is far from fully understood.^14^, ^15^, ^16^ Except for genetic risk factors, other acquired risk factors include physical inactivity, smoking, education, diet, high blood pressure, traumatic brain injury, type 2 diabetes, cerebrovascular diseases, and mental status.^17^ In the following contexts, AD refers mainly to LOAD.

Based on genome‐wide association study (GWAS) analysis, AD is described as a multiplex model that is not only challenged by aging, immune response and inflammation, amyloidosis, tau accumulation, and cholesterol, but also newly enriched by possible mechanisms like endocytosis, ubiquitination (ie, the posttranslational modification of adding ubiquitin molecules to lysine residues), and vascular alterations. All or some of these deficits processes together trigger the AD disease cascade, which leads to neuron defects observed in the AD process.^14^

### 1.1 Pathology and general theories

The pathogenesis of AD is related to the formation of extracellular aggregates of beta‐amyloid (Aβ) plaques and the intracellular accumulations of neurofibrillary tangles (NFTs) consisting of the hyperphosphorylated microtubule‐associated protein tau.^18^ Aβ is generated by protease cleavage of β‐amyloid precursor protein (APP) which is a kind of type Ⅰ transmembrane protein.^19^ Three families of secretases with different biological functions are related to APP cleavage. Potential α‐secretases include several “a disintegrin and metalloproteinase” (ADAM) family members: ADAM9, ADAM10, and ADAM17,^20^ while β‐site APP cleaving enzyme 1 (BACE1) is the main β‐secretase in the brain.^21^ Normally, γ‐secretase refers to a complex which is comprised of 4 core components: PSEN1 and 2, nicastin (NCSTN), anterior pharynx defective 1 (APH1), and presenilin enhancer‐2 (PEN2).^22^

In disease state, pathological alterations in the brain mainly start from anomalous cleavage of APP by β‐secretase and γ‐secretase^23^, ^24^ to generate insoluble Aβ fragments which is known as the amyloidogenic pathway.^18^ First, full‐length APP is cut at the N‐terminus by β‐secretase, and then, the sAPPβ ectodomain is released outside of the cell membrane while a 99 amino acid carboxy‐terminal fragment (β‐CTF or C99) is retained inside.^25^ This is the first and rate‐limiting step that removes the majority of extracellular portion of the APP.^18^ The β‐CTF or C99 is further cleaved by the γ‐secretase at different sites and generates amyloid peptides among which Aβ_40_ and Aβ_42_ are two major species according to their chain length in the brain.^26^ Compared to Aβ_40_, Aβ_42_ is less abundant, insoluble, and neurotoxic and it is supposed to play a key role in Aβ plaques accumulation.^18^, ^23^ While in EOAD Aβ accumulation can be explained by the mutations in proteins responsible for APP metabolism (APP, PS1, and PS2) favoring Aβ_42_ cleavage, in LOAD the mechanisms determining amyloidogenic switch are multifaceted and based on increased β‐secretase expression, altered trafficking (APOE, CLU, SORL1), or degradation (PICALM, SORL1, CD33, BIN1, CD2AP, ABCA7).^27^ The abundance of Aβ in the micro‐environment has been reported to contribute to the activation of several kinases (glycogen synthase kinase 3 (GSK3β),^28^ adenylate kinase 1 (AK1),^29^ and CDK5), which regulate the phosphorylation process of tau protein.^30^ This phosphorylation is highly regulated by those activated kinases. Once hyperphosphorylated, the tau proteins tend to be unstable and aggregate to large chunks of tau filaments and form NFTs.^31^ The ratio of Aβ_40/42_, phosphorylated tau (p‐tau), and total tau (t‐tau) in CSF are core hallmarks of AD.^18^ However, AD is a chronic progressive degenerative disease, and its pathogenesis is the result of many factors.^32^ Other hypotheses include cholinergic hypothesis,^33^ inflammatory hypothesis,^34^ mitochondrial cascade hypothesis,^35^ and neurovascular hypothesis.^36^

### 1.2 The role of microglia cells

Neuroinflammation has become a major contributing factor in AD's development. It includes multifarious inflammatory events of the central nervous system (CNS) under pathological conditions. Chronic inflammation dysregulates the clearance of misfolded tau proteins through cellular and molecular influence. This abnormal clearance of neuro proteins leads to synaptic dysfunction, which eventually results in the occurrence and progression of AD.^37^ In this process, the activation of microglia is the key element of neuroinflammation.^34^ Recently, it has been shown that microglial activation and tau accumulation appear simultaneously and colocalize in the living human brain, which crucially proved that the activation of microglia is not just a result of disease progression, but rather a key upstream mechanism for AD's progression.^38^

Under physiologic conditions, microglia act as auxiliary cells contributing to homeostasis throughout life. As immune sentinels, microglia are constantly surveying their environment for pathogens or other stressors and scavenge apoptotic debris or dysfunctional synapses.^39^ In the presence of immune stimuli, microglia switch to an immune‐activated state characterized by increased phagocytosis and increased expression of cytokines, receptors, and other inflammation‐related chemokines.^40^ If this response exceeds or becomes chronic, it may lead to irreversible damage to the surrounding tissue and to neurodegenerative diseases.^40^ On the other hand, insufficient or no microglial reaction to minimal stimuli also can have a detrimental effect and may end up in neurodegenerative disease. The seminal studies of McGeer and coworkers first reported on a large number of HLA‐DR‐positive reactive microglia and significant plaques in the brains of AD patients.^41^ In fact, a set of molecular control mechanisms, referred to as microglial checkpoints,^42^ prevent exceeding microglial immune responses.

Four major checkpoint pathways are known to restrain microglial immune activity under physiologic conditions:

1. *Seclusion from the blood circulation:* Microglia are secluded from the blood circulation by the blood‐brain barrier (BBB).^43^, ^44^, ^45^
2. *Soluble factors:* Various soluble factors influence the immune profile of microglial cells. A crucial role in microglial cell homeostasis plays transforming growth factor‐β (TGF‐β), a cytokine highly present and abundantly secreted in the steady state.^46^ Other soluble factors like the anti‐inflammatory cytokines interleukin‐(IL‐)4 and IL‐13 adopt protective roles in particular during injury or inflammation.^47^ For example, IL‐13 serves as a negative feedback mechanism for activated microglia cells. Activated microglial cells increase their IL‐13 expression upon lipopolysaccharide (LPS) injection in rat brains leading to cell death of immune‐activated microglial cells.^48^ Furthermore, microglia influence their immune phenotype by the expression of neurotransmitter receptors (eg, for ATP, adenosine, GABA, adrenaline or acetylcholine) rendering them highly responsive to the neighboring neurons and their created neurotransmitter milieu.^49^
3. *Cell‐to‐cell interactions:* Direct cell‐to‐cell interactions with neighboring neurons have been demonstrated to downregulate microglial immune activity.^50^ One of this immune‐restraining mechanism is mediated by the receptor‐ligand pair, fractalkine receptor (CX_3_CR1) on the microglial cells and its ligand CX_3_CR1, expressed on neuronal cells.^51^ Other receptors such as CD200R are also associated with inhibitory signaling pathways.^52^
4. *Transcriptional regulators:* Transcriptional factors or for example the chromatin modifiers Mef2C and MeCP2 are further able to regulate microglial immune response and activity.

The impact of these microglial checkpoints alters in the course of aging and becomes deeply counterproductive in the sequelae of chronic neurodegenerative disease.

*During brain development* neuronal cells are being developed in oversupply and much of them go into apoptosis. The same is true for nascent synaptic connections. Microglial cells act regulatorily in both directions by releasing cytokines supporting growth and differentiation of neuronal progenitor cells and by removing apoptotic cells and pruning superfluous synapses.^53^, ^54^, ^55^ Inhibitory checkpoints not only prevent microglia from mounting exceeding responses to immune stimuli but also orchestrate microglial functions toward the changing needs of the developing brain. Several molecules (TGF‐β, MeCP2, and CX_3_CR1) are crucial for these early microglial activities.^56^, ^57^, ^58^

*During adulthood*, microglial phagocytosis of apoptotic bodies and synaptic pruning continues, albeit on a low level. In addition to TGF‐β, MeCP2, and CX_3_CR1, now MafB plays a significant role. Lack of MafB within microglial cells leads to increased expression of interferon and inflammation‐related genes. Interestingly, lack of MafB has no impact in the fetal or newborn mice brain.^59^

**FIGURE 1:**
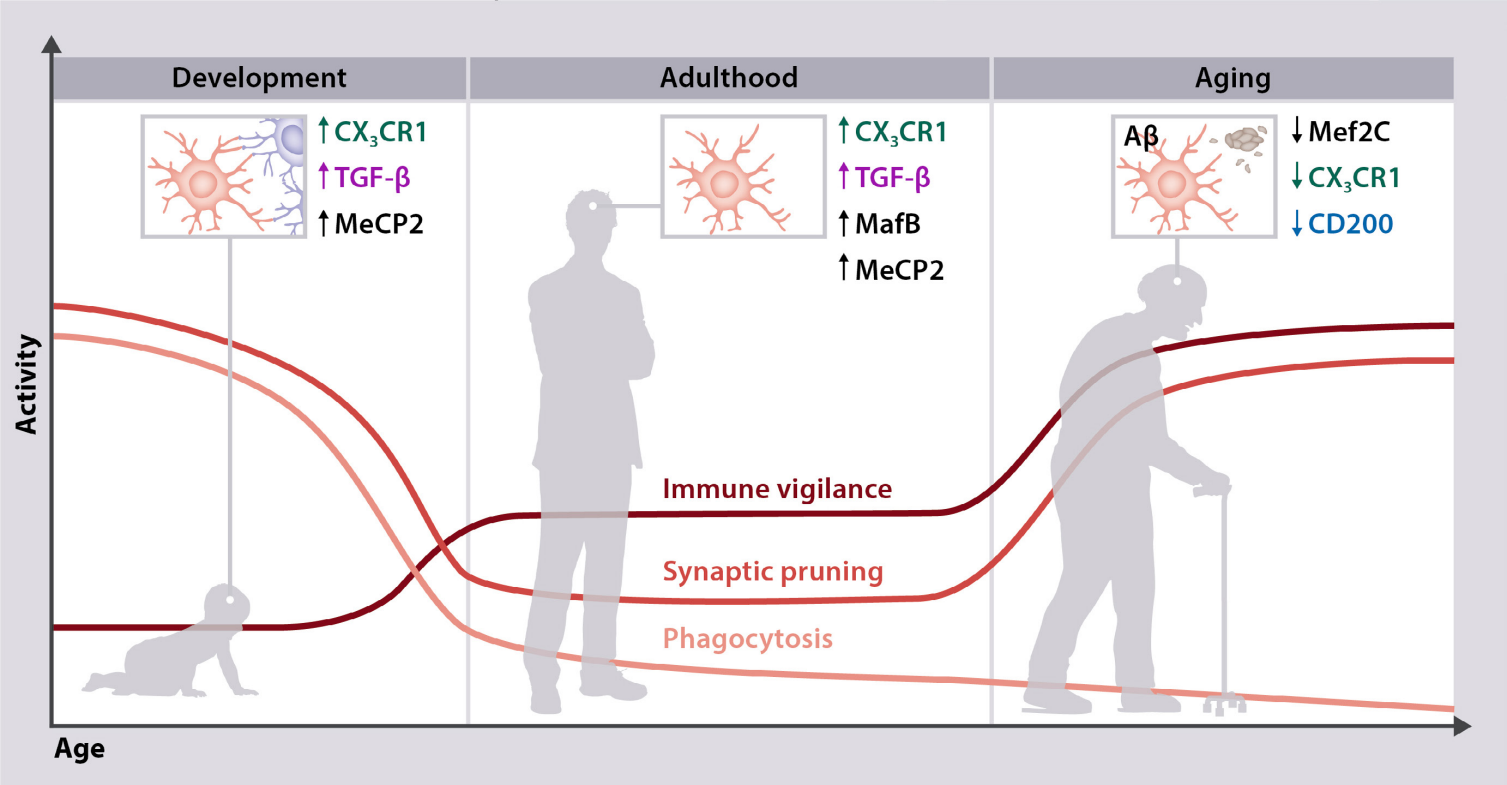
Microglial main functions, that is, immune vigilance, synaptic pruning, and phagocytosis and their level of activity undergo a circle throughout life. The checkpoint mechanisms help to regulate these alterations. Adapted by permission from: Springer Nature, Nature Neuroscience, Deczkowska et al. 2018.^42^

With aging, microglia undergo profound changes affecting their homeostasis and switching their phenotype from synaptic modulators at childhood, to resting in adulthood and activated in the old human brain (Figure 1). In the old human brain both immune‐related molecules and toxic compounds amass. This is related to impaired microglial functioning itself, but also to a changing neuronal environment. Aging microglial cells express more inflammatory‐related molecules. Synaptic pruning is increased while the phagocytic activity decreases. The neuronal environment produces more microglial activators and less microglial checkpoint molecules. Accordingly, chronic interferon‐(IFN)‐I expression, exceeding secretion of neurotransmitters, release of ATP from dying cells, or amyloid‐β (Aβ) plaques may further contribute to a phenotypic shift of microglial cells toward an immune‐activated and dysregulated cell.^60^ And yet, adequately working microglial checkpoints are able to counteract these signals^42^ (Figure 1). This scenario is acerbated in AD in which overactivated microglia by releasing inflammatory cytokines perpetuate Aβ production, while failing in plaque removal.^61^

### 1.3 Inflammaging and immunosenescence

Although inflammaging and immunosenescence are mostly mentioned in one breath, they describe different phenomena. Senescence is associated with cells that stop dividing thereby entering a state of permanent growth arrest but still being highly metabolically active.^62^, ^63^ Cells in various tissues undergo aging processes both as a naturally occurring cell fate and as responses to environmental stressors that evoke cellular damage over time. Overall, immunosenescence loosely describes the declining functioning and resilience of the immune system resulting in higher incidence of infections and diseases which in turn enhances the level of proinflammatory cytokines. There is not one exact cytokine or biomarker characteristic for senescent cells, and it remains debatable whether immunoscenescence is the cause or a consequence of aging itself. Regardless, the secretion of cytokines, growth factors, proteases from senescent cells represents the senescence‐associated secretory phenotype (SASP).^64^, ^65^ One of those is IL‐6, typically elevated in elderly individuals.^66^ At this point, immunosenescence is closely intertwined with inflammaging which generally describes the state of elevated levels of proinflammatory mediators and low‐grade inflammation.^66^ Inflammaging results from the uncontrolled activation of the innate immune response, provoked by an increasing antigen accumulation throughout life. Senescent cells gradually switch from oxidative phosphorylation toward glycolysis producing only 2 ATPs for their energy supply even in the presence of oxygen. The senescent cell is further linked to increased ADP and AMP relative to ATP.^67^, ^68^ These metabolic changes further aid that the immune system slowly becomes dysregulated and cellular responses less efficient. A decline in cellular stress response capacity disturbs the redox balance leading to augmented oxidative stress, genotoxic damage, and accelerated telomere attrition, as is observed during replicate senescence.^69^ Upon stimulation with an antigen, senescent immune cells are thus not able to mount a fast and strong response but rather prefer a slower pathway.^70^

Pioneering work on the mechanisms of aging has been done on yeasts (*Saccharomyces cerevisiae*), worms (*Caenorhabditis elegans*), and insects (*Drosophila melanogaster*) demonstrating that there exist evolutionary‐conserved pathways to respond to for example oxidative, radiation‐induced, or thermal stress, which might have a considerable impact on the longevity of organisms.^71^, ^72^, ^73^, ^74^ In humans, more powerful mechanisms to counteract stress developed, such as inflammation and innate immunity with macrophages being in the spotlight and early on stage. The direct relation between age and macrophage activation, macroph‐aging, was then referred to as inflamm‐aging. It has to be pointed out that the biological effects of stress and the ensuing adaptive responses depend on the severity of the stressor and an individual's ability to cope with it.^75^, ^76^ The two‐hit hypothesis of inflammaging postulates that the inflammaging evolves over time and represents the first hit as an inflammatory background. A second hit, however, is required in order to initiate disease and disability such as AD or atherosclerosis. The interplay between environmental factors and individual genetic makeup thereby determines the extension of longevity and the ability to modulate the aging rates.^77^

Host‐pathogen interactions during a lifetime are at the origin of low‐grade chronic inflammation propagating to the brain through permissive organs such as the olfactory or visual systems or through a leaking blood‐brain barrier (BBB). Furthermore, aging alters the components of innate immunity ranging from the expression of signaling molecules to the behavior of neutrophils, monocytes, dendritic cells, NK cells, etc.^78^ One recent study reports a unique set of peripheral mediators in plasma, sIL‐6R, TIMP‐1, and sTNFR‐I, that when considered in aggregate inversely correlate with the dementia onset.^79^ In addition, infiltrating peripheral immune cells, such as CD4^+^ and CD8^+^ T cells, etc, are enriched in AD brains.^80^ Peripheral type 1 and type 17 T‐helper (Th1, Th17) cells have been reported to be associated with releasing of inflammatory cytokines in multiple AD mouse models^81^, ^82^ and also human subjects. These data support that peripheral inflammatory events in adult life set the stage for neuroinflammation with aging.^79^

With respect to inflammation in the mouth, experimental gingivitis studies comparing young (<25 years) and elderly individuals (>65 years) revealed that both groups formed comparable amounts of biofilm during a 3‐week period of refraining from oral hygiene, however elderly subjects developed more pronounced signs of inflammation, a denser inflammatory infiltrate, higher levels of IgG, and plasma cells but lower polymorphonuclear (PMN) cells than their younger counterparts.^83^, ^84^, ^85^

### 1.4 Association AD – periodontal disease

Periodontitis and severe periodontitis are considered the 6th and 11th most prevalent chronic condition in the world.^86^, ^87^ Data from the National Health and Nutrition Examination Survey (NHANES) over the years 2009/2010 and 2011/2012 showed that 46% of US adults had periodontitis, with 8.9% suffering from severe periodontitis.^88^ For the global burden of severe periodontitis, even a higher prevalence of 11% was reported.^86^, ^88^ For the population over 65 years, there may be a seven‐fold higher risk of periodontitis compared to adults between 30 and 34 years. The prevalence of periodontitis in the 70‐ to 81‐year‐old age group has risen and it is likely to further increase given the expanding segment of the elderly population.^89^

The etiology of periodontitis lies in the presence of a dysbiotic biofilm, the host‐biofilm interaction, and a predisposition of the host.^90^ Periodontitis may thereby act as a driver for a chronic immune response. The dissemination of bacteria and systemically elevated inflammatory cytokines might further be a risk factor for the onset or progression of chronic diseases such as for AD. For example, elderly adults suffering from more severe periodontal disease had higher CRP plasma levels than those with mild forms of the disease.^91^ A retrospective study evaluated the data of 262 349 participants out of the Korean National Health Insurance Screening Cohort. In comparison with non‐periodontitis participants, those with chronic periodontitis had an elevated risk for overall dementia (adjusted hazard ratio = 1.06; 95% CI = 1.01‐1.11) and AD (aHR = 1.10; 95% CI = 0.98‐1.22).^92^ Recently, a cohort study reported, that patients with chronic periodontitis for at least 10 years had a higher risk of developing AD, but also had a higher prevalence of hyperlipidemia, depression, traumatic brain injury, and co‐morbidities than non‐periodontitis patients.^93^ Another cohort study showed that severe periodontitis and the periodontal inflamed surface area were associated with an increased incidence of mild cognitive impairment (MCI) in community‐dwelling individuals over a follow‐up period of 5 years.^94^

## 2 ORAL MICROORGANISMS IN AGED PEOPLE

Although recent decades have seen a profound change in the age pyramid of the society with a steadily expanding segment of the elderly population over 65 years of age, the number of studies evaluating the bacterial composition of the microbiome in the aging mouth is still limited. It has, however, to be kept in mind that the human oral microbiome is highly diverse sheltering an estimated number of 700 bacterial species. Age‐related changes, eg, the reduction of salivary flow, systemic comorbidities, multiple medications, or insufficient oral care are likely to impact the composition of the oral microbiome and the appearance of oral diseases.

### 2.1 Oral microbiota

Studies on the oral microbiome in elderly people can basically be divided into two groups; one group of studies focused on elderly people 65 years of age or older; the other group of studies recruited residents of nursing homes most of them suffering from multiple health and cognitive impairments.

The results from the first group of studies are summarized in Table 1. Generally, the microbial load increased with age.^95^ Individuals who exhibited a low number of lactobacilli, *Streptococcus mutans,* and yeasts had better general oral health than those who presented with high bacterial counts.^95^ While *Aggregatibacter actinomycetemcomitans* seemed to be found less frequently in elderly individuals than in adults under 25 years of age,^96^, ^97^, ^98^ *streptococci* and *lactobacilli* were more frequently and in higher numbers detected in elderly individuals.^99^ An investigation on saliva and supragingival biofilm samples of 79 dentate individuals (divided into four subgroups according to their age, 20‐39, 40‐59, 60‐79, >80 years of age) found no differences among age groups in relation to total counts of bacteria in saliva, as well as for the prevalence of Streptococcus mutans and Spirochaetes species. However, Actinomyces species, especially Actinomyces naeslundii and Actinomyces oris, were found in higher proportions in the supragingival biofilm of subjects over 60 years of age.^100^ No difference was detected in the prevalence of *S* *mutans* and *Spirochaetes* species, *Actinomyces* species predominated in the group >80 years of age. Yeasts were correlated with increasing age and with dentures.^101^ *Staphylococcus aureus*, enteric rods, and *Candida albicans* correlated with the presence of dentures but not with general health.^102^ Feres et al analyzed microbial samples of three different age groups concluding on no substantial differences in bacterial numbers or proportions across the age groups. Only a trend was discerned toward higher proportions of *Fusobacterium nucleatum* subspecies. In patients with refractory periodontitis, elderly individuals revealed higher numbers of enteric rods and *Pseudomonas* species while younger adults showed higher counts of *staphylococci*.^103^ A recent microbiome analysis of subgingival biofilm showed a high abundance of *Streptococcus*, *Leptotrichia wadei,* and *Rothia denticariosa* in individuals aged 65 years and more. The bacterial diversity was higher in individuals with periodontitis than in periodontally healthy ones.^104^ With severity of periodontitis, *Sneathia amnii*‐like sp, *Peptoniphilaceae* [G‐1] bacterium HMT, *Porphyromonas  gingivalis*, *Fretibacterium fastidiosum*, certain *Treponema* ssp increased; however, *P gingivalis* ranked 89th and *Tannerella forsythia* 73rd with an abundance of 0.25% and 0.32% in severe periodontitis.^104^

**TABLE 1:** Studies on oral microbiota in elderly people aged 65 years and more

Author, Country, Year	Inclusion criteria	Study: design, number of participants, groups	Samples, microbiological methods	Results
Holm‐Pedersen et al, Denmark, 1975^83^	Good oral health, no active periodontal disease, caries and defective restorations, a minimum of natural teeth	Experimental gingivitis in young (20‐24 y; n = 10) and elderly (65‐78 y; n = 11) individuals, abstain from oral hygiene during 21 d	Plaque from the crevice area of the labial surfaces of teeth, Gram‐staining (microscopy)	Plaque accumulation, gingival inflammation, and exudate higher in the elderly group, no difference in morphology of the microorganisms between groups
Slots et al, USA, 1990^96^	Periodontitis, not medically compromised, no periodontal or antibiotic treatment within the preceding 3 mo, ≥3 sites with PPD of ≥6 mm	Total n: 1624; 15‐89 y	Subgingival biofilm samples, culture	*Aggregatibacter actinomycetemcomitans* higher prevalence (74%) in patients <25 y than in adult or geriatric patients, *Prevotella intermedia* (prevalence 45%) not related to age
Slots et al, USA, 1990^103^	Refractory periodontitis patients, no periodontal or antibiotic treatment within the preceding 2 mo, ≥3 sites with PPD of ≥6 mm	Total n: 3075; 12‐95 y	Subgingival biofilm samples, culture	Older subjects higher counts of enteric rods and *Pseudomonas* sp than younger individuals, younger individuals more staphylococci, detection of yeasts not related to sex or age
Fure and Zickert, Sweden, 1990^99^	Agree to participate	Total n: 208 Cross‐sectional study, age groups 55, 65 and 75 y‐olds were examined	Resting and stimulated whole saliva, pooled plaque samples	Increase with age: total counts of microorganisms, *S* *mutans*, *Streptococcus sobrinus*, lactobacilli
Percival et al, UK, 1991^105^	≥7 teeth (including 1 molar), absence of active oral disease, no dentures; no recent antimicrobial therapy or other drug therapy, including immunosuppressives, no diabetes	Total n: 79, Age groups: (A) 20‐39 y, n = 30; (B) 40‐59 y, n = 23; (C) 60‐79 y, n = 16; and (D); ≥80 y, n = 10	Supragingival molar plaque and unstimulated whole saliva, culture	Total microbial counts no difference in saliva between age groups, although lowest count in group D, lactobacilli in saliva higher in groups C and D than in group A, of staphylococci, yeasts highest in groups C and D
Savitt et al, USA, 1991^97^	≥1 site with 2 mm attachment loss, no antibiotic therapy in the previous 6 mo	Total n: 3142 sites from 1492 subjects divided into groups according to age with intervals of 10 y	Subgingival plaque samples, DNA probes	*Aggregatibacter actinomycetemcomitans* highest in the youngest age group (10‐19 y), *Porphyromonas gingivalis* lowest at 10‐19 y and highest in the age groups 30‐39 and 40‐49 y
Beck et al, USA (North Carolina) 1992^106^	Age ≥65 y	366 Black and 297 White community‐dwelling adults	Plaques samples from the molar region, IF	Prevalences of *A actinomycetemcmoitans*, *Pr intermedia,* and *P*. *gingivalis* higher in blacks than whites. *Porphyromonas gingivalis* was found in 38.8% of Blacks and 9.4% of Whites. Subjects with *P*. *gingivalis* or *Pr intermedia* had a higher prevalence of sites with LA ≥7 mm
Marsh et al, UK, 1992^101^		120 healthy subjects (41 with partial dentures). 4 age groups: 20‐39; 40‐59, 60‐79, >80 y	Molar plaque and saliva, culture	Total microbial counts, presence of yeasts and lactobacilli in saliva and plaque higher in partial‐denture wearers. Yeasts and lactobacilli increase in saliva with age, enterobacteria occasionally present in saliva of denture wearers in group >80 y
Drake et al, USA, 1993^107^	Age ≥65 y	Longitudinal study including 375 Blacks and 300 Whites aged >65 yfrom the Piedmont Health Survey	Subgingival plaque samples, IF	*Porphyromonas gingivalis* and *Pr intermedia* were more frequently associated with each other than with *A* *actinomycetemcomitans, S* *mutans*, lactobacilli Blacks were more frequently infected and more severely infected with *P gingivalis* and *Pr intermedia* than Whites
Ohman et al, Sweden, 1995^102^	Age = 79 y; participants selected on a statistical basis	100 persons (47 men and 53 women; 75 with dentures)	Samples from palatal mucosa, and the right angulus oris, or from dentures, culture	*Staphylococcus aureus* in 5 patients, *Enterobacteriaceae* spp. in only 1 individual. *Candida albicans* not found in palatal mucosa samples from individuals without dentures; Prevalence of *Staphylococcus aureus*, and *C albicans* did not correlate with general health
Ohazama et al, Japan, 2006^108^	Age >80 y	Comparative study, Total n = 60; independently living n = 22; in mean 24.7. teeth), nursing home n = 38, in mean 4.2 teeth	Swabs from the vestibular areas of the maxilla and mandible, culture	Higher counts of *Staphylococcus* sp and *C albicans* in the nursing group than in the independent group
Preza et al, Norway, 2009^109^	No signs of oral mucosal disease, or root caries and had no antibiotic use up to 1 mo prior to sampling	N = 30 (n = 8 residents of a long‐term care facility), age 73‐93 y, mean number of teeth 23 (range 9‐36)	Swabs from tongue dorsum, buccal fold, hard palate, supra‐ and subgingival plaque from the same root surface, microarray	A total of 175 species and clusters Microbiota diverse, more site‐ than subject‐specific Most commonly detected species: *Streptococcus oralis* oral taxon 707, *Veillonella atypica* oral taxon 524, *Streptococcus parasanguinis* oral taxon 721 and *Fusobacterium nucleatum* subsp *polymorphum* oral taxon
Al‐Haboubi et al, UK 2014^95^	Age ≥60 y, ≥6 teeth, living independently in the community, no antibiotics the last 4 wk	Total n: 186 mean age 70.2 y Age groups: 60‐64, 65‐74, 75‐84, ≥85 y)	Saliva samples, culture	Younger age groups (60‐64; 65‐74 y) fewer decayed root surfaces, lower plaque index and lower lactobacilli counts than those ≥85 y. Low levels of lactobacilli, mutans streptococci and yeasts in saliva correlated with better overall oral health
Feres et al, Brazil/USA, 2016^98^	>20 y old and in good general health; subjects with periodontitis ≥15 natural teeth and 4 teeth with ≥1 site each with PPD and CAL ≥5 mm; periodontally healthy individuals ≥20 teeth, no sites with PD or CAL >3 mm, and <20% of the sites with bleeding on probing. Exclusion criteria: pregnancy, nursing, periodontal therapy and antibiotics within the previous 3 mo, any systemic condition that might affect progression of periodontitis, need of antibiotics for routine periodontal procedures	Analyses of cross‐sectional and longitudinal studies at the University São Paulo and at the Forsyth Institute, Boston from 1999 to 2014, including data from 1330 subjects, 1084 with periodontitis and 246 with periodontal health Three age groups: <35 (young), 35‐64 (adults), and >64 y (older adults)	Samples of subgingival bacterial biofilm; Checkerboard analysis for 40 bacterial species	No statistically significant differences in the levels or % of the evaluated bacterial species evaluated between the age groups; older adults higher levels by trend of *F nucleatum* subspecies Periodontitis: more *P gingivalis* and *Tannerella forsythia*, *A actinomycetemcomitans* in the young group than in the other 2 groups; *Actinomyces naeslundii* and *Actinomyces oris* highest in the oldest group Differences between health and periodontitis for the proportions of the evaluated bacterial species very similar among the three age groups
Meyer et al, Switzerland, 2019^110^	Aged ≥70 y, partially edentulous with titanium implants, no active periodontitis or peri‐implantitis, no heavy smokers and individuals with systemic diseases. No antibiotic or anti‐inflammatory therapy for the previous 2 mo	Experimental mucositis: 20 partially edentulous participants with titanium implants refrained oral hygiene for 3 wk, then after professional self performed oral hygiene 3 wk. Mean age 77.0 ± 5.7 y	Subgingival biofilm at preselected tooth or implant sites over a period of 6 wk, qPCR for 6 species	*Parvimonas micra* most frequently, *Treponema denticola* the least detected microorganism. No differences between teeth and implants No detection of *A actinomycetemcomitans*, similar profiles at teeth and implants, only *P micra* higher at implants than teeth at 3 wk without oral hygiene, prevalence of *P gingivalis* increase from baseline of about 30%) to more than 75% at 6 wk
Jiang et al, China, 2019^111^	>60 y, ≥20 natural teeth, good oral hygiene, no other oral infectious diseases, no removable partial dentures, no bridge or implant, no systemic diseases, no antibiotic use within 2 mo	Microbiome analysis including 92 saliva and plaque samples of 24 caries patients and 22 healthy controls	Microbial DNA was extracted, Next generation sequencing	Samples represented 16 phyla, 29 classes, 49 orders, 79 families, 149 genera, and 305 species. Predominant phyla: *Proteobacteria, Bacteroidetes, Firmicutes, Fusobacteria, Actinobacteria*, and *Saccharibacteria*. The co‐occurrence analysis of the predominant genera revealed significant interactions among oral microbiota and exhibited more complex and aggregated bacterial correlations in caries‐free groups
Papapanou et al, USA, 2020^104^	WHICAP Ancillary Study of Oral Health recruited 1130 participants aged >65 y. their periodontal status could be classified according to the CDC/AAP criteria	Cross‐sectional cohort study, 739 participants, mean age 74.5 y, 32% were male, 3.5% current smokers	Periodontal status CDC/AAP criteria; From dentate individuals 4 biofilm samples from the most posterior tooth; Next Generation Sequencing	20.6% periodontally healthy, 54.5% moderate and 22.4% severe periodontitis Most abundant taxa: Streptococcal species, *Leptotrichia wadei*, and *Rothia dentocariosa* consistently among the most abundant taxa; *T denticola* ranked third in abundance in severe periodontitis, all other established periodontal pathogens not among the highest ranked abundant species (eg, *T forsythia* ranked 73rd; *P gingivalis* 89th, *A actinomycetemcomitans* ranked 261st)

Abbreviations: CAL, clinical attachment loss; CDC/APP, Centers for Disease Control and Prevention/ American Association of Periodontology; IF, immunofluorescent assays; LA, loss of attachment; PPD, pocket probing depth; qPCR, quantitative polymerase chain reaction; WHICAP, Washington Heights‐Inwood Columbia Aging Project.

A few studies included only individuals living in nursing homes. Differences in microbiological profiles were found when participants were divided in groups with and without dentures. Dentate patients without dentures had the highest counts of *F nucleatum*, *C albicans* was more present in edentulous patients wearing dentures, whereas *P gingivalis* counts were associated with the presence of teeth in denture wearing individuals.^112^ Stays in hospitals affected the amount and composition of oral microbiota. In bedridden patients staying in hospital for more than 3 months, the oral biofilm contained in a high percent of the patients *Enterobacter cloacae*, *Klebsiella pneumoniae,* MRSA, *Pseudomonas aeruginosa*, *Streptococcus agalactiae*, and *Stenotrophomonas maltophilia*.^113^ A subgroup of studies examined the oral microbiota of nursing home residents with symptoms of aspiration pneumonia. The presence of an aspiration pneumonia was found to be associated with the presence of *P gingivalis* in the dental biofilm, (OR 4.2, 95% CI = 1.6, 11.3); and *Streptococcus sobrinus* (OR 6.2, 95% CI = 1.4, 27.5) and *S* *aureus* (OR 7.4, 95% CI = 1.8, 30.5).^114^ In most studies, data on the provided oral hygiene were not reported but this aspect cannot be neglected. But when residents received professional oral health care in weekly intervals as did Ishikawa et al^115^ and Adachi et al,^116^ both clinical parameters, that is, PPD and viable counts of *C albicans* and *S aureus* significantly decreased over the course of 5‐6 months compared to the control groups.

Taken together, the available data suggest that aging increases the load of oral microorganisms but affects mainly the composition of the oral microbiota. Factors responsible for microbial dysbiosis seem to be systemic diseases, wearing of dentures, living in nursing homes and no access to adequate oral hygiene measures.

## 3 STUDIES IN HUMAN ON THE ASSOCIATION PERIODONTAL MICROORGANISM AND AD

Several studies in humans investigated the presence of oral bacteria in brain samples with respect to AD (Table 2). The study that elicited most discussions was published by Dominy et al.^117^ The percent of brain samples with positive results for *P gingivalis* most important virulence factors argine‐ and lysine‐specific gingipains (Rgp and Kgp) was very high, in particular in patients with AD, there, 90% and more were positive for both Rgp and Kgp. In postmortem analysis of Parkinson disease patients, *P gingivalis* was identified but not *T forsythia* or *Treponema denticola*.^118^ However, this is not consistent with other findings. The first report on postmortem analysis of brains found positive results for certain *Treponema* spp. but not for *P gingivalis*.^119^ Thereafter, Poole et al reported positive results for *P gingivalis* LPS but not for *Treponema* sp or *P gingivalis* gingipains.^120^ In another analysis,^121^ *P gingivalis* was detectable, but it was not the most prominent species. Emery et al found more bacterial reads in AD than in cognitive healthy controls but periodontal bacteria were not identified.^122^

**TABLE 2:** Studies in humans on the association periodontal microorganisms and Alzheimer disease

Author, year, country	Study: design, participants, groups	Samples and analyzed variables	Results
Reviere et al, USA, 2002^119^	16 donors with AD and 18 con	Brain tissue samples Different oral *Treponema* sp by using PCR or monoclonal antibodies	*Treponema* detected in 14 AD and 4 con by PCR, most present *T socranskii*, *Treponema denticola,* and *Treponema pectinovorum* (no detection of *Porphyromonas gingivalis*, *Aggregatibacter actinomycetemcomitans*, *Eikenella corrodens,* or *Prevotella intermedia*) Detection of *Treponema* sp at trigeminal nerves
Kamer et al, USA, 2009^124^	Cross‐sectional Total n = 34 AD = 8 Con = 16 (Age: 40‐>80 y)	Plasma IgG against *A actinomycetemcomitans*, *P gingivalis* and *Tannerella forsythia*; plasma TNFα, IL‐1β, and IL‐6	IgG against bacteria: more positive results in summary (bacteria not presented specified) TNFα higher in AD, no difference IL‐1β and IL‐6 (but trend to lower IL‐6 in AD)
Sparks Stein et al, USA (Kentucky), 2012^128^	Longitudinal study, follow‐up, started in 1989, annual controls Total n = 158 Age at baseline in mean ≈72 y At follow‐up (mean ≈12 y thereafter) AD = 35 MCI = 46 Con = 77	APOE genotype; serum IgG against *A actinomycetemcomitans*, *P gingivalis*, *T forsythia*, *T denticola*, *Fusobacterium nucleatum*, *Pr intermedia,*and *Campylobacter rectus*	Baseline: significant difference MCI, AD each vs Con: APOE ε4, MMSE AD vs Con: higher IgG against *F nucleatum*, *Pr intermedia* at baseline and after conversion, MCI vs Con: higher IgG against *F nucleatum*, *C rectus*, *Pr intermedia* at baseline, lower levels against *P gingivalis*, *T denticola, C rectus,* and *T forsythia* after conversion
Poole et al, UK 2013^120^	Total n = 20 AD = 10 (63‐85 y) Con = 10 (22‐103 y)	Brain specimens immediately post‐mortem *P gingivalis* LPS and gingipains, *T forsythia*, *T denticola* (immunofluorescence)	4 / 10 AD samples (age 83‐85 y) positive result for *P gingivalis* LPS, Con samples negative AD and Con samples, all negative for *P gingivalis* gingipain, *T forsythia, and T denticola*
Noble et al, USA (State of New York) 2014^129^	Case‐cohort study, follow‐up for 5 y Total n = 219 AD = 110 Con = 109	Serum IgG against *P gingivalis*, *T forsythia*, *A actinomycetem‐comitans*, *T denticola*, *C rectus*, *Eubacterium nodatum*, and *Actinomyces naeslundii* genospecies‐2	High IgG against *A naeslundii* (0.640 ng/mL, present in 10% of subjects) was associated with increased risk of AD; high anti‐*E nodatum* IgG (0.1755 ng/ml; 19% of subjects) was associated with lower risk of AD
Emery et al, UK, 2017^122^	AD = 12 Con = 10	Post‐mortem brain samples 16SrDNA analysis	More bacterial reads in AD than Con, more Propionibacteria, no detection of bacteria associated with periodontal disease (contamination not completely excluded)
Laugisch et al, Germany/Switzerland, 2018^130^	Pilot study Total: n = 40 AD = 20 DEM‐noAD = 20 age ≤70 y, early changes in AD pathology	Clinical oral indices; selected bacteria (serum, CSF, subgingival biofilm); selected pro‐inflammatory cytokines (serum, GCF); IgG against *A actinomycetemcomitans*, *P gingivalis*, *T socranskii/denticola* (serum, CSF) t‐tau, Aβ_1‐42_ (CSF)	Worse periodontal indices in both groups, possible intrathecal immune response against selected bacteria in 7 AD and 9 DEM‐noAD; in AD t‐tau level associated with both serum IgG against *P gingivalis* and MCP‐1/CCL‐2
Liu et al, China, 2019^132^	Total: n = 78 AD = 39 Con = 39	APOE genotype; microbiome analysis of saliva	Microbiome: lower richness and diversity, more *Moraxella*, *Leptotrichia*, *Sphaerochaeta*, less *Rothia* in AD; AD: APOE4^+^(n = 23) less *Actinobacillus* and *Actinomyces*
Bennett et al, USA, 2019^121^	AD = 10 Con = 9	Brain tissue (frontal cortex); RNA, transcriptome aligned against different microglial genes, *Toxoplasma gondii*, *Trichinella* sp, *Babesia microti*, *Borrelia burgdorferi*, *T denticola*, *P gingivalis*	Microorganisms: highest % of reads *B microti*; less reads for *B burgdorferi*, *P gingivalis,* and *T denticola*; no significant difference AD – Con, *B burgdorferi* (*P* = .067), and *P gingivalis* (*P* = .068) approached significance Microglial genes: more expression of complement C1q subcomponent subunit B precursor (C1QB) and less expression of purinergic receptor P2Y12 (P2RY12)
Dominy et al, USA, 2019^117^	Brain tissue samples For RgpB and Kgp total n = 212 AD = 110 (age: 51‐97 y) Con = 102 (age: 19‐81 y) Saliva and CSF total n = 10 (all AD)	Brain tissue samples: IHC, IF, WB, for RgpB and Kgp, qPCR for *P gingivalis* Saliva and CSF: qPCR for *P gingivalis*	51/53 AD samples positive for RgpB 49/54 AD samples positive for Kgp Both load higher than in Con (18/46) positive RgpB, 26/50 positive Kgp, each RgpB, and Kgp correlated with AD diagnosis, tau load Other samples, colocalization of RgpB with neurons, but not microglia, tau tangles, and intraneural Aβ Brain lysates of cerebral cortex: 3/3 AD positive for Kgp (WB), *P gingivalis* (qPCR) 5/6 C positive for Kgp, *P gingivalis* (qPCR) CSF: 7/10 AD positive for *P gingivalis* (except for 1 [72 y] all 59‐62 y) Saliva: 10/10 positive for *P gingivalis*
Bathini et al, Switzerland, 2020^131^	Cross‐sectional cohort study Total n+ 80 (mean age ≈70 y) AD = 17 MCI = 21 ConR = 15 Con = 27	Saliva samples: microbiome analysis; selected pro‐inflammatory cytokines; APOE genotype (no oral clinical data)	Decrease of *Filifactor villosus* associated with disease severity; MCI vs other groups: depletion of number of bacterial taxa, depletion of *P* *gingivalis*, *Filifactor alocis*, *Prevotella tannerae*; increase of *Cardiobacterium vallarum*, *Leptotrichia wadei*; increase of cytokines
Beydon et al, USA, 2020^126^	Samples and data from NHANES‐CMS: n = 6650	Clinical oral indices, Serum IgG against 19 bacteria	IgG against *P gingivalis* correlated with AD, against *A actinomycetemcomitans* inverse correlation, correlation with IgG against *C rectus* in the age 55+ y
Beydoun et al, USA, 2020^127^	Samples and data from NHANES‐CMS: included n = 1439 (n = 768 with clinical periodontal data) AD = 277 (166) All DEM = 549 (294)	Serum IgG against 19 periodontal bacteria and *Helicobacter pylori*; cognitive impairment, demographic data, nutrition, race, clinical oral indices	55% of samples positive for *H pylori*, positive correlation with certain *Prevotella* ssp, *C rectus* with respect to AD incidence, positive association CAL, PD, and *H pylori* serum IgG
Franciotti et al, Italy, 2021^125^	Cohort study Total n = 78 ND = 21 (8 AD), mean age 70.6 y noND = 28, mean age 67.6 y Con = 29, mean age 56.1 y	Clinical oral indices, counts of *P gingivalis* in tongue brushes, serum IgG against *P gingivalis*	Clinical oral data comparable in ND and noND groups but inferior to Con *P gingivalis* counts highest in ND group, IgG against *P gingivalis*, no difference between ND and NoND
Kamer et al, USA/Sweden, 2021^134^	Cross‐sectional study Total n = 48 (all cognitive healthy, mean age 69.2 y)	Clinical periodontal indices; CSF: levels of A*β*42, p‐tau181=>grouping by using a cutoff, APOE genotype, subgingival biofilm microbiome analysis	A*β*42^+^ (n = 18) significantly higher dysbiotic index than A*β*42‐(n = 24) Results not so striking regarding p‐tau
Yang et al, USA, 2021^135^	Longitudinal observational study Total n = 68 MCI (AD confirmed) = 34 Con = 34	Microbiome analysis of oral soft tissue swabs, plasma CRP and LPS CSF for t‐tau and p‐tau, inflammatory markers; APOE genotype	No difference in α‐diversity, after adjustment for ApoE4 more *Pasteurellacae* and less *Lautropia mirabilis* in MCI, IL‐17, and IL20RA in CSF negatively associated Shannon index; IL‐1α, TSLP, IL10RA, and IL13 in CSF negatively associated with *Pasteurellacae*
Patel et al, Canada, 2021^118^	Postmortem study of Parkinson disease 44 prefrontal cortex control samples (males, 44‐97 y)	*P gingivalis* sequence reads	10/44 samples positive for *P gingivalis*, no positive result for *T forsythia* and *T denticola* In *P gingivalis* + samples gene upregulation of genes expressed in the anterior hypothalamic area, genes encoding ribosomal proteins, ER translocation genes
Wu et al, Taiwan, 2021^133^	Total n = 35 AD = 17 Con = 18	Dental biofilm samples microbiome analysis, oral health data (DMFT)	Decreased microbial diversity in AD; no difference of DMFT; more Firmicutes in AD; *Fusobacteria* and *Porphyromonas* decreased in AD; *Lactobacillus*, *Streptococcaceae*, *Actinomycetaceae,* and *Veillonella* enriched in AD

Abbreviations: AD, Alzheimer disease; Con, healthy controls; ConR, healthy controls at risk; CSF, cerebrospinal fluid; DEM‐noAD, dementia not AD; DMFT, decayed/missing/filled teeth; ER, endoplasmic reticulum; MCI, mild cognitive impairment; MMI, mild memory impairment; ND, neurodegenerative disease; noND, neurological disease, not neurodegenerative; WB, Western blot.

These reports appear to suggest that *P gingivalis* or its gingipains may enter the brain. However, the published data raise the question on the quality of the obtained samples (time and storage after death of the patients). Early postmortem, bacteria can invade very fast the tissues of the body since physiological barriers do not function anymore.^123^ The often non‐adequate oral hygiene, and the high prevalence of periodontitis, are related to high bacterial load of bacteria associated with periodontitis in the oral cavity. Of further interest are the results by Poole et al who found positive signals for *P gingivalis* LPS but not for gingipains,^120^ which is in contrast to the findings of Dominy et al.^117^ However, when interpreting the data, sensitivity and specificity of the used methods need to be discussed.

Several studies measured serum or plasma IgG against bacteria associated with periodontal disease. Kamer et al^124^ reported higher IgG levels against *P gingivalis*, *T forsythia* and *A* *actinomycetemcomitans* in AD patients compared to subjects without cognitive impairment. Nevertheless, these data are also not consistent. Franciotti et al did not see differences in IgG levels against *P gingivalis* in patients with neurodegenerative diseases despite higher *P gingivalis* counts on tongue.^125^ Beydon et al^126^ reported a positive correlation of AD with IgG against *P gingivalis* and an inverse against *A* *actinomycetemcomitans*. There was a positive correlation of IgG against certain periodontal bacteria with those against *Helicobacter pylori*.^127^ In a longitudinal study, Sparks Stein et al^128^ found higher IgG against *Prevotella intermedia* and *F nucleatum* in individuals developing AD at follow‐up vs cognitive healthy individuals; however IgG against *P gingivalis* decreased in the patients developing AD after conversion. Noble et al showed high IgG against *Actinomyces naeslundii* being associated with increased risk for AD.^129^ In our pilot study comparing AD and dementia patients without AD, we failed to detect differences in serum IgG against *A* *actinomycetemcomitans*, *T denticola/socranskii*, *T forsythia,* and *P gingivalis*.^130^ But t‐tau level in cerebro‐spinal fluid of AD patients was associated with both serum IgG against *P gingivalis* and monocyte chemoattractant protein‐1/CC‐chemoligand‐2 (MCP‐1/CCL‐2).^131^

Microbiome analyses of saliva samples were published two times. In a study from Switzerland, the MCI group showed a depletion of numbers of bacterial taxa and of *P gingivalis*, *Filifactor alocis* vs the other groups (healthy control, controls at risk, AD group).^132^ Another analysis of saliva samples obtained from each 39 AD and healthy controls found less bacterial richness and diversity in the AD group, among the genera the relative abundance of *Moraxella* was higher in AD than in controls, whereas for the other most abundant 29 genera (among them *Porphyromonas*) no differences were found.^133^ In both studies, no oral clinical data are reported; therefore, the microbiome findings cannot be related to the number of teeth or periodontal disease status.

A recently published study on biofilm microbiome data confirmed the decreased microbial diversity in AD. At phylum level, more Firmicutes were quantified in AD than in controls, at genus level *Fusobacterium* and *Porphyromonas* were decreased, *Lactobacillus*, *Streptococcaceae*, *Actinomycetaceae,* and *Veillonella* were enriched in AD vs control.^134^ Although the authors presented data on DMFT (decayed/missing/filled teeth) index, no data on periodontal disease were recorded. Subgingival biofilm microbiome analysis in cognitive healthy individuals stated a correlation of dysbiosis (defined as an increase of periodontal bacteria) with the detection of β‐amyloid in cerebrospinal fluid (CSF).^135^ At genus level *Fretibacterium*, *Prevotella* and *Dialister* were increased, whereas *Corynebacteria*, *Actinomyces,* and *Capnocytophaga* were decreased.^136^ Interestingly, in that analysis, periodontal disease severity did not differ between CSF β‐amyloid‐positive and ‐negative individuals.^137^ A microbiome analysis of oral soft tissue samples did not find a difference in diversity related to cognitive status, but there was an inverse correlation of Shannon‐index with CSF IL‐17 concentration.^138^

A study marginally touching the association with AD was published by Yussof et al.^139^ Reported microbiome data associated with AD were combined with other databases to analyze pathways how high levels of alcohol cause a shift in oral microbiome and its importance in the development of AD. Several genera, *for example, Dialister*, *Clostridium*, *Eubacterium*, *Bacteroides*, *Porphyromonas*, *Shigella*, *Salmonella*, *Fusobacteria* were linked with AD. Overlapping species of AD and alcoholics were *Porphyromonas* spp. and *Neisseria* spp.^136^ Overall, the data available on the oral microbiome of AD subjects so far appear not consistent among studies, suggesting a lack of methodological consensus guidelines when studying microbial dysbiosis in association with chronic disease. This demands that investigations in the future use defined criteria, such as number of teeth, periodontal disease status, frequency of dental hygiene, co‐morbidities, nutritional regimen when analyzing, and interpreting the oral microbiome. In addition, humoral and inflammatory responses in the cohorts would allow to understand how pathogen‐host interaction change in the course of the disease, casting light on the potential causal association between oral dysbiosis and AD conversion.

## 4 PORPHYROMONAS GINGIVALIS – ANIMAL AND IN VITRO‐STUDIES

### 4.1 Animal studies

In autopsy studies, *P gingivalis* was the most frequently identified periopathogen. This microbe is known for being capable of activating, but also subverting the host immune reaction and of invading host tissue cells and is therefore regarded as the keystone‐pathogen of periodontal disease.^137^ The major virulence factors of *P gingivalis* in a neuroinflammatory context are the lipopolysaccharide (LPS) and the gingipains Rgp and Kgp proteases. LPS is a component of the outer bacterial cell membrane and capable in stimulating a pro‐inflammatory reaction in host cells by activating toll‐like receptors (TLR) 2 and 4. Gingipains are essential for aggregation and co‐aggregation within the biofilm; they enable invasion in the host tissue and also evade the host immune reaction by cleaving and inactivating cytokines, IgG, and factors of the complent system.^138^, ^139^ In the current literature, 19 studies that investigated the impact of *P gingivalis* infection on the progression of AD in animal models were identified (Table 3).

**TABLE 3:** Studies using animals on the association *Porphyromonas gingivalis* – Alzheimer disease

Model (animal)	Experimental setting	Results	Ref.
6 wk old T2DM mice (ApoE^−/−^)	*P gingivalis* 3/wk over 16 wk, Con: vehicle and *F nucleatum*	Hippocampus: microgliosis ↑, astrogliosis ↑, tau ↑, TLR4, IL‐6R, IL‐1R2, NFκB ↑ Compared to *F nucleatum*: astrogliosis and TLR4 n.s.	Bahar et al 2021^143^
4 wk old male rats	*P gingivalis* (ATCC49417, HG184, W50, and W50 non‐encapsulated), palatal injection, twice, Con: sham	Cognitive: spatial memory, hits ↓, latency ↑, observed/expected distance ↑ Systemic: *P gingivalis,* RgpA, Kgp, IL‐1β, IL‐6, IFN‐γ, TNF‐α ↑ Hippocampus: *P gingivalis*, RgpA, Kgp, IL‐1β, IL‐6, INF‐γ, Aβ42, MDA, p‐tau ↑CSF: *P gingivalis,* RgpA, Kgp, IL‐1 ↑, Aβ_42_ (IL‐6 ↑ only in W50), (TNF‐α ↑ only in HG184)	Diaz‐Zuniga et al 2020^146^
4 and 12 wk old female mice	*P gingivalis*, oral administration, 3/wk, 6 wk, Con: sham	4 wk old animals: n.s. vs Con 12 wk old animals: Cognitive: escape latency ↑, crossing times ↓ in Morrison water maze Brain: IL‐1β, IL‐6, TNF‐α ↑	Ding et al 2018^147^
44 wk old mice	*P gingivalis*, oral administration, daily, 6 wk, COR286 (Rgp inhibitor) COR271 (Kpg inhibitor), moxifloxacin Con: sham, Rgp, or Kgp knock‐out	Brain: Aβ_42_ ↑, not in Rgp or Kgp knock‐out Gad67 pos interneurons ↓ Effects were reversible via COR271, COR286, moxifloxacin, or a combination after infection	Dominy et al 2019^117^
12 mo old female mice	*P gingivalis* LPS, intraperitoneal injection	Cognitive: latency retention ↓ Cortex: IL‐6, IL‐17 ↑ Iba1 pos microglia: IL6, IL‐17 ↑, Nissl pos neurons: IL6, IL‐17 n.s., Aβ_42_ ↑ (pos rel. with microglia IL‐17)	Gu et al 2020^148^
6 and 13 mo old APP/PS1 transgenic mice	*P gingivalis* LPS, intracerebroventricular injection, one (6 mo old) or continuous (6 and 13 mo old), Con: vehicle, littermate	Periventricular: migration of Iba‐1 and CD3 pos cells ↑, Aβ_40_, Aβ_42_, NF‐κB, COX‐2 n.s. The impact of aging was more relevant than the infection	Hayashi et al 2019^145^
10 wk old rats	*P gingivalis* LPS, oral administration, 3/wk, additional TAK‐242 (TLR4 antagonist), Con: saline	Cognitive: latency ↓, t spent in target quadrant, crossing times ↑ in Morris water maze Systemic: Plasma: IL‐1β, IL‐6, IL‐8, IL‐21, LPS ↑; PBMC: TLR4, CD14 ↑; Cortex: microgliosis ↑, astrogliosis ↑, IL‐1β, IL‐6, IL‐8, IL‐21, LPS ↑, APP, APLP1, APLP2, BACE1, PS2, Aβ_42_, APP, Aβ1‐40/Aβ1‐42, Tau ↑, ADAM10 ↓, TLR4, CD14 ↑, IRAK1, p65, pp65 ↑ Effects reversible via TAK‐242	Hu et al 2020^153^
6 wk old mice	*P gingivalis*, oral administration, 3/wk, 22 wk Con: vehicle	Hippocampus: gingipain in 9/9 samples, located in microglia, astrocytes and neurons IL‐6, IL‐1β, TNF‐α ↑, APP, BACE1 ↑, ADAM10 ↓, PSEN1 n.s., Aβ_42_ ↑, p‐tau ↑ microgliosis ↑, astrogliosis ↑, intact neurons ↓	Ilievski et al 2018^152^
62 wk old APP transgenic mice	*P gingivalis*, oral administration, 5 wk, Con: vehicle	Hippocampus: Aβ_40_ ↑, Aβ_42_ ↑ cortex: Aβ_40_ ↑, Aβ_42_ ↑ brain: IL‐1β ↑, TNFα ↑, endotoxins ↑	Ishida et al 2017^144^
12 wk old mice	*P gingivalis*, intraperitoneal injection 2/wk, 3 wk or gavage, additional Salvianolic acid B (BACE1 inhibitor), daily 4 wk, Con: vehicle	Cognitive: escape latency ↑, t spend in target quadrant ↓, crossing times ↓ in Morris water maze, spontaneous alternation index ↓ in Y‐maze Hippocampus: IL‐1β, IL‐6, ROS, MDA ↑, SOD, GSH‐Px ↓, Aβ_40_, Aβ_42_ ↑, APP, BACE1, PS1 ↑, ADAM10 ↓	Liu et al 2020^149^
12 mo old mice	*P gingivalis*, intraperitoneal injection 3/wk, 3 wk, Con: saline	Systemic: liver: mRNA TLR2, IL‐1β, APP, CatB ↑, BACE1, ACE n.s.	Nie et al 2019^151^
8 wk old T2DM mice (ApoE^−/−^)	*P gingivalis*, *Treponema denticola*, *Tannerella forsythia* or polymicrobial, oral lavage, 12 or 24 wk, Con: sham	*P gingivalis* positive in 6/12 brain samples (12 wk) and in 9/11 (24 wk) in monoinfected *P gingivalis* mice, in 2/12 (24 wk) in polyinfected mice An invasion could not be shown for *T denticola* and *T forsythia*	Poole et al 2015^140^
6 mo APP/PS1 transgenic male mice	*P gingivalis* LPS, interpapillary injection with or without ligature, once, 8 wk, Con: no injection	Cognitive: escape latency ↑, t spent in target quadrant ↓, discrimination index ↓ Hippocampus and Cortex: Aβ_42_ ↑, APP, BACE1 ↑, ADAM10↓ microgliosis ↑, astrogliosis ↑, iNOS, COX‐2, IL‐1β, TNF‐α ↑ Ligature aggravated the results	Qian et al 2021^155^
10 wk old TNFα transgenic mice and T2DM mice (ApoE^−/−^)	*P gingivalis*, oral administration, 12 wk Con: sham	Hippocampal capillaries: protein carbonisation ↑, pyramid: protein carbonisation ↑, damaged neurons ↑	Rokad et al 2017^141^
10 wk old T2DM mice (ApoE^−/−^)	*P gingivalis*, oral administration, 24 wk	Age‐related granules and appeared with more widespread IgG in cerebral capillaries	Singhrao et al 2017^142^
8 wk old male mice	*P gingivalis*, oral administration, 3/wk, 24 wk	Hippocampus: Aβ_40_ ↑, Aβ_42_ ↑, BACE1, PS1, PS2 ↑	Su et al 2021^162^
8 wk old rats	*P gingivalis*, intravenous injection, 3/wk, 4 and 24 wk, Con: vehicle	Hippocampus: IL‐1β, IL‐6, TNFα ↑, astrocyte activation ↑, PP2A activity ↓ 12 wk: p‐tau181, pTau231 ↑, p‐tau 181 ↑ compared to 4 wk	Tang et al 2021^154^
2 and 12 mo old CatB heterozygous mice	*P gingivalis* LPS, daily, 5 wk, Con: vehicle and CatB homozygous mice	2 mo old: n.s. vs Con 12 mo old: Hippocampus: TRL4 ↑, CatB ↑ in microglia and neurons, not in astrocytes IL‐1β, TLR2 ↑ in microglia, not in neurons and astrocytes	Wu et al 2017^150^
15 mo old female mice	*P gingivalis*, intraperitoneal injection, 2/wk, 3 wk, Con: saline	Cognitive: latency ↓ at 3 wk Hippocampus: CatB, RAGE, CD31 pos cells ↑, NFκB ↑, Aβ42 ↑ around CD31 pos cells, pos correlated to RAGE	Zeng et al 2021^163^

Abbreviations: ACE, angiotensin converting enzyme; ADAM10, a disintegrin and metalloproteinase domain‐containing protein 10; APLP, amyloid β precursor‐like protein; ApoE^−/−^, apolipoproteine E deficient; APP, amyloid β precursor protein; BACE1, β‐site of APP cleaving enzyme; CatB, cathepsin B; CD, cluster of differentiation; Con, control; COX‐2, cyclooxygenase‐2; CSF cerebrospinal fluid; Gad67, glutamate decarboxylase 67; GSH‐Px, glutathione peroxidase; I iNOS, inducible nitric oxide synthase; Iba‐1, Ionized calcium‐binding adaptor molecule‐1; Kgp, lysine gingipain; LPS, lipopolysaccharide; MDA, malondialdehyde; n.s., non‐significant; NFκB, nuclear factor κ‐light‐chain‐enhancer of activated B cells; n.s., not significant; PBMC, peripheral blood mononuclear cell; PP2A, protein phosphatase 2A; PS, presenilin; p‐tau, phosphorylated tau; RAGE, receptor for advanced glycation end products; RgpA, arginine gingipain A; ROS, reactive oxygen species; SOD, superoxide dismutases; t, time; T2DM, type 2 diabetes melitus.

In addition to wild‐type or rodent models, type 2 diabetes mellitus mouse models with a gene inactivation for apolipoprotein E (ApoE^−/−^)^140^, ^141^, ^142^ or leptin receptor (db/db)^143^ or transgenic models overexpressing APP^141^, ^144^ or even APP and PSEN1 (5XFAD)^145^ were infected with *P gingivalis* or *P gingivalis* LPS. The infection was administrated by either oral administration, intraperitoneal, or even intracerebroventricular injection.

With regard to cognitive impairment, studies showed that recurring applications of *P gingivalis* or *P gingivalis* LPS deteriorated the spatial learning and memory in behavioral experiments like Morris water maze.^145^, ^146^, ^147^, ^148^, ^149^, ^150^ This declining cognitive performance occurred only in middle‐aged animals (10 weeks or older); when both young (4‐8 weeks) and middle‐aged animals were included, there was no cognitive impairment in the young animals.^147^, ^150^

Sustained *P gingivalis* or *P gingivalis* LPS administration resulted in a systemic immune reaction with increased pro‐inflammatory cytokines, like IL‐1β, IL‐6, TNF‐α, among others, which is consistent with periodontal animal models in general. In terms of AD specific markers, Nie et al^151^ showed that an intraperitoneal injection of *P gingivalis* increased the mRNA expression of AβPP770, an amyloid β protein precursor in the liver tissue, whereas the expression of the converting enzyme (BACE1) was not significantly different to the livers of the vehicle treated control group.

Of overriding interest were the potential effects in the brain. *Porphyromonas gingivalis* was identified in the animals' brains after oral administration.^140^, ^142^, ^143^, ^146^, ^152^ The capability of overcoming the BBB and entering the brain tissue was strain‐specific^146^ and oral *P gingivalis* infection was accompanied by protein carbonization in the hippocampal capillaries.^141^ Histologically, chronic *P gingivalis* or *P gingivalis* LPS administration increased the number of activated microglial cells and astrocytes in the cortex^148^, ^153^ and in the hippocampus^143^, ^145^, ^150^, ^152^, ^154^ as an indicator of the local inflammation. Pro‐inflammatory mediators, like IL‐1β, IL‐6, TNFα, INF‐γ, were elevated compared to controls on mRNA and protein level.^143^, ^144^, ^146^, ^147^, ^148^, ^149^, ^150^, ^152^, ^153^, ^154^

In terms of the β amyloid deposition, precursor proteins (APP) were elevated after *P gingivalis* or *P gingivalis* LPS infection.^152^, ^153^ BACE1, which cleaves APP and, therefore, is essential for the amyloid β deposition was elevated in the hippocampal region.^149^, ^152^, ^153^ Compared to the untreated controls, infected animals showed an elevated Aβ_40_ and Aβ_42_ deposition after various ways of administration.^117^, ^144^, ^146^, ^148^, ^149^, ^152^, ^155^, ^156^

Another hallmark of AD is the tauopathy. The levels of phosphorylated tau protein (p‐tau) were increased after *P gingivalis* or *P gingivalis* LPS infection when compared to the controls.^144^, ^146^, ^152^, ^153^ A higher abundance of neurofibrillary tangles was also observed by immunofluorescence.^152^

Some of the studies showed signs of neuroinflammation in the hippocampal area after intracerebroventricular injection of *P gingivalis* or *P gingivalis* LPS. This may not be surprising when stating a functional immune system of the infected animals. And this is not a unique property of *P gingivalis*, because cognitive impairment and inflammatory reaction could also be shown for LPS originating from other bacteria.^157^, ^158^, ^159^, ^160^, ^161^ Taken together, the results of the animal studies demonstrated that a recurrent infection with *P gingivalis* or *P gingivalis* LPS led to cognitive impairment and local inflammatory reaction in the hippocampal region consistent with a progression of AD. These changes did mainly occur in elder, transgenic, or knock‐out animals and could be alleviated by specific inhibitors.

### 4.2 In vitro studies

One major aspect in the etiology of AD is the deposition of amyloid‐β plaques in microglial cells. The amyloid‐β is generated not only locally in the brain tissue, but also in the periphery.^164^ For an intracerebral accumulation, Aβ needs to transcend the BBB. Experiments using a brain microvascular endothelial cell line (hCMEC/D3) demonstrated that an infection with *P gingivalis* resulted in a TLR4‐ and NFκB/cathepsin B (CatB) ‐dependent increase of the receptor for advanced glycation end products (RAGE) mRNA compared to the untreated control. The transcellular transport of Aβ_42_ across a single layer of endothelial cells was elevated in the following.^164^ In a study by Liu et al, monocyte (THP‐1) and macrophage cell lines (RAW 264.7), correspond to a systemic infection, were co‐incubated with *P gingivalis* LPS. The monocyte/ macrophage conditioned medium was then transferred to a primary leptomeningeal cell culture (model for CSF‐blood barrier). This resulted in an increased expression of IL‐1β and TNF‐α.^165^ Conditioned medium was capable in activating neuron‐precursor‐cells derived astrocytes indicated by an elevated level of glial fibrillary acidic protein (GFAP) demonstrated by immunofluorescence staining.^166^

Microglial cell lines (BV‐2, MG6, SV40) and a macrophage/monocyte cell line (RAW 264.7) were infected with *P gingivalis* LPS. The cell lines were activated and entered a pro‐inflammatory state via the TLR2/4 induced NFκB/ STAT3^167^ or NFκB/ CatB pathway^151^ and showed signs of oxidative stress characterized by increased levels of inducible nitric oxide synthase.^166^ The expression of pro‐inflammatory mediators like IL‐1β, pro‐IL‐1β, IL‐6, IL‐8, IL‐17 was elevated compared to the untreated controls.^148^, ^151^, ^166^, ^167^ Experiments using conditioned medium demonstrated similar effects.^166^ In another study performed by Ishida et al,^144^ an enhanced production of IL‐1β and TNF‐α could be demonstrated after incubation of microglial cell lines with *P gingivalis* LPS together with Aβ_42_, but not for Aβ_42_ or *P gingivalis* LPS alone.

Additionally, SV40 cells showed an increased expression of CD86, a specific, costimulatory ligand in T‐cell activation.^166^ Regarding the accumulation of Aβ, *P gingivalis* LPS infection resulted in a higher mRNA expression of TREM2. The function of TREM2 in Aβ deposition remains still unclear, but it is supposed to be involved in the intracellular uptake and the degradation of Aβ.^168^ Furthermore, a co‐cultivation of macrophages with *P gingivalis* resulted in an elevated level of Aβ precursor proteins.^151^

The invasion and intracellular persistence of *P gingivalis* in neuronal precursor cell (NPC) ‐derived neurons was visualized in transmission electron microscopy images.^169^ The bacteria were found bond to the cell membrane or internalized in lysosomes. *Porphyromonas gingivalis* turned in a viable, but not cultivable status although the proteolytic activity of Kgp and Rgp remained active during the experimental period. The authors furthermore observed an elevated abundance of autophagic vacuoles, a pathohistological trait of AD neurons, a loss in synapses and an increase of p‐tau.^169^ The level of Aβ precursor proteins and of CatB were increased after incubation with conditioned medium, but not with *P gingivalis* LPS.^148^ Whereas Ishida et al found an increased deposition of Aβ_40_ and Aβ_42_ in neuronal cell cultures after incubation with *P gingivalis* LPS.^144^ IL‐1β in conditioned medium induced a higher phosphorylation of tau proteins and a decreased activity of protein phosphatase 2 A (PP2A) than the untreated control.^154^ PP2A is the principal dephosphorylating enzyme and loss in function is crucial in the development of tauopathies.^170^, ^171^

In summary, many in‐vitro and animal studies underline the potential of *P gingivalis* to induce AD‐related alterations. In general, these alterations could only be induced in susceptible animals and were not evident in wild‐type controls. In animals, often a direct application into the brain that is not closely related to the clinical situation was used. Moreover, controls were most sham or no bacteria. Finally, comparison with other periodontal bacteria (eg *Treponema* ssp, *T forsythia*, *F nucleatum*), should be considered to determine a potentially specific role of *P gingivalis*.^162^

## 5 OTHER ORAL MICROORGANISMS – IN VITRO AND ANIMAL STUDIES

Studies in humans failed to reveal consistent results on an association of *P gingivalis* and AD. Thus, in‐vitro and animal research on other oral microorganisms is also of interest. The few reports on these aspects are summarized in Table 4. In mice infected with *P gingivalis* or *T denticola* over a period of 24 weeks, both bacteria could breach the BBB and induce the accumulation of β‐amyloid.^162^

**TABLE 4:** In‐vitro studies or studies using animals on the association oral bacteria (not *Porphyromonas gingivalis*) – Alzheimer disease

Investigated species	Model	Experimental setting	Results	Ref.
*Aggregatibacter actinomycetemcomians* (LPS from serotypes a, b, and c)	Cell culture (rat mixed hippocampal cells and mixed astrocyte/microglia cultures)	Treatment of cells with each 50 ng/ml LPS for 48 h Expression of cytokines, TLR2, TLR4 Level of Aβ_1‐42_ Cell morphology of neurons in hippocampal cultures	mRNA microglia IL‐1β ↑↑(b), IL‐4 ↑↑(a), IL‐6 ↑↑ (b), IL‐10 ↑↑(a) ↑(c), IL‐17 ↑↑ (b), TNF‐α ↑↑ (b), TLR2 ↑(a), ↑↑(c) TLR4: ↑↑(a,b) ↑(c) mRNA hippocampal IL‐1β ↑↑ (b), IL‐4 ↑(a), IL‐6 ↑↑ (b), IL‐10 ↑(a), IL‐17 ↑↑ (b), TNF‐α ↑↑ (b), TLR2 ↑↑(b) TLR4: ↑(a) Secretion hippocampal cells IL‐1β ↑(b), IL‐6 ↑(b), TNF‐α: ↑(b), Aβ_1‐42_: ↑(a) ↑↑(b,c) Cell morphology: changes after b (but no change in viability)	Diaz‐Zuniga et al 2019^172^
*Aggregatibacter actinomycetemcomians* (extracellular RNA in OMVs)	Animal (6‐wk old male mice)	Intracardiac injection of OMVs, staining after 4 h and 24 h for OMVs and RNA	At 4 h, OMV and RNA in blood vessels, at 24 h spreading crossing of blood brain barrier, induction of TNF‐α	Han et al 2019^173^
*Fusobacterium nucleatum*	In‐vitro (In‐vivo)		Production of amyloid‐like FadA mainly in stationary growth phase Presence of amyloid‐like FadA in periodontitis	Meng et al 2021^174^
*Treponema denticola* (*Porphyromonas gingivalis*)	Animal (8‐wk old male mice)	Continuous infection with *P gingivali*s or *T denticola* over 24 wk, thereafter brain samples of mice analysis for bacteria; amyloid precursor protein (APP), Aβ_1‐40_ and Aβ_1‐42_ and enzymes involved of degradation (BACE1, PS1, PS2)	Hippocampus: 7/10 positive for *P gingivalis*; 7/10 positive for *T denticola* Aorta samples : 9 positive *P gingivalis*, 7 *T denticola* Trigeminal ganglion samples: 3 positive *T denticola* both bacteria induced Aβ_1‐40_ and Aβ_1‐42_ accumulation by upregulation of BACE1 and PS1	Su et al 2021^162^

Abbreviations: APP, amyloid β precursor protein; BACE1, β‐site of APP cleaving enzyme; FadA, Fusobacterium adhesin A; LPS, lipopolysaccharide; OMV, outer membrane vesicle; PS, presenilin; TLR, toll‐like receptor.

Stimulation of rat brain cells with *A* *actinomycetemcomitans* LPS resulted in a serotype‐specific upregulation of inflammatory cytokines and toll‐like receptors (TLR)‐2 and 4.^172^ *Aggregatibacter actinomycetemcomitans* serotype A LPS increased the expression of the anti‐inflammatory cytokine IL‐10.^172^ In hippocampal cells, a secretion of β‐amyloid was induced, mainly by LPS of serotype b and c.^172^ Extracellular RNA in outer‐membrane vesicles of *A* *actinomycetemcomitans* is able to cross the BBB and to induce inflammation.^173^ *Fusobacterium nucleatum* produces an amyloid‐like adhesion.^174^

The ability of bacterial DNA to promote Tau misfolding and aggregation was analyzed in an in vitro study.^175^ Largest promoting effects were observed for *Escherichia coli* and *Tetzerella alzheimeri*, moderate effects were induced by *P gingivalis* and *Borrelia burgdorferi*, whereas no significant effect occured after addition of human DNA, *C albicans* DNA, or DNA from *P aeruginosa* and *Tetzosporium hominis*.^175^ (*T alzheimeri* was first described by the authors, it belongs to *Brucellaceae* and was isolated from a periodontal pocket of a patient with AD.^175^)

## 6 GUT MICROBIOME AND AD

Outside of the oral cavity, certain associations between microbiota and the etiopathogenesis of AD are under discussion. For example, *B burgdorferi*, a spirochete causing Lyme disease,^176^ was cultivated from cerebrospinal fluid obtained from patients with AD,^177^ and this bacterium was identified postmortem within senile plaque consisting of β‐amyloid.^178^

Furthermore, gut dysbiosis has been documented through stool analysis in AD progression^179^ and has been shown to aggravate the pathology in Drosophila models.^180^ The gut microbiome appears to be of particular interest since it has the most abundant and diverse community in the body. While the composition of the gut and the oral microbiota is unique, important connections that could play a role in the development of several diseases might be present.^181^ In the pathogenesis of AD, the gut‐microbiome‐brain axis has also been discussed.^182^ The microbiome‐gut‐brain axis functions by three pathways, via the enteric nervous system, via the circulation and the blood‐brain barrier by gut microbe metabolites and via the modulation of the immune system by microbial‐associated molecular pattern (MAMPs).^182^ A few times stool samples of AD patients were compared with those obtained from cognitively healthy controls. Once a decrease of α‐diversity was described,^183^ in another study there was no difference in α‐diversity between AD and the respective controls.^184^ The composition of gut microbiota differs between cognitively healthy individuals and AD patients. But reported data are not consistent. *Enterococcaceae* were found increased in one analysis^183^ and in the other decreased^184^ in AD patients vs cognitively healthy individuals. Decreased numbers of *Enterococaceae* in AD were reported also in another analysis, but related to the APOE genotype, individuals with at least one APOε4 allele (AD and cognitive healthy) harbored higher *Enterococaceae* counts in the gut microbiome compared to the others.^184^ This raises the question if there is an influence by the life style (food intake) of AD patients. Thus, a study in patients with newly diagnosed AD or MCI vs healthy controls might be of interest. Here, it was shown that *Bacteroides*, *Lachnospira,* and *Ruminiclostridium*_9 decreased, whereas *Prevotella* increased.^185^ The importance of the gut microbiome is further underlined by a case report where due to a *Clostridium difficile* infection a fecal microbiota transplantation (FMT) was made in a 90‐year‐old AD patient whose cognitive abilities improved thereafter.^186^ The FMT changed the gut microbiota to a higher abundance of *Bacteroidales*, *Bacteroidea*, *Tannerellaceae*, and *Actinobacteria*.^186^

As mentioned before, transgenic mice are used to verify etiologic and supportive factors in the development of AD. APP/PS1 transgenic mice do not react differently to wild‐type mice aged 3 months, but they develop impaired spatial learning and memory when being 6 months old, and these changes aggravated at 8 months.^187^ In the transgenic mice but not in the wild‐type mice Aβ‐plaque in brain were identified.^187^ The α‐diversity of the gut microbiome did not change over time in wild‐type mice, whereas it decreased in APP/PS1 transgenic mice of older age.^187^ At family level, there was a higher abundance of the families *Helicobacteriacae* and *Desulfovibrionacae*; at genus level *Helicobacter*, *Odirobacter* were more and *Prevotella* abundance was less in APP/PS1 mice than wild‐type mice^187^ ; however, *Prevotellaceae* increased with age in the gut of APP/PS1 mice.^187^ The role of the gut microbiome in development of Aβ amyloid plaque in brain might be underlined by the fact that in susceptible (male APP/PS1‐21 transgenic) mice depletion of the gut microbiome by an antibiotic mixture of five antibiotics resulted in a reduction of Aβ amyloid plaque in the brain, whereas application of a single antibiotic did not reveal significant changes.^188^

EFAD mice are transgenic mice that overexpress h‐APOE3 (E3FAD mice) or h‐APOE4 (E4FAD mice), gut microbiome analysis found no difference in α‐diversity between these mice but a higher relative abundance of *Anaeroplasma* and lower relative abundance of *Prevotella*, *Ruminococcus,* and *Sutterella* in EFAD4 mices than in EFAD3 mices.^189^ Also, the study by Tran et al confirmed no difference in α‐diversity between human transgenic homozygeous APOE3‐TR and APOE4‐TR mice, at phylum level *Deferribacteres* were more and *Candidatus Saccharibacteria* were less abundant in APOE4‐TR than in APOE3‐TR mice.^190^ FMT obtained from 5xFAD mice to a wild‐type mice induced memory impairment together with decreased hippocampus neurogenesis, elevated release of proinflammatory cytokines in colon, plasma and microglia, which did not occur after FMT from a wild‐type mice.^191^ After FMT, the gut microbiota differed, at genus level increased levels of *Rikenella* and decreased levels of *Prevotella* and *Barnesiella* were found in mice, which received FMT from 5xFAD mice compared to normal mice.^191^

All experiments show the importance of the gut microbiota related to AD but underline a genetic susceptibility. In wild‐type mice, an 18‐month infection with *H pylori* or *Helicobacter felis* always caused gastric inflammation but did not induce the formation of amyloid plaques or neuroinflammation in the brain.^192^ Eight‐week‐old triple‐transgenic AD and their respective wild‐type control mice were exposed to a cocktail of nine probiotic bacterial strains for 4 months.^193^ In AD‐mice with exposure to probiotics vs non‐exposed AD mice, cognitive decline decreased together with reduced Aβ aggregates, plasma concentration of inflammatory cytokines, modifications of gut hormones and gut microbiota; wild‐type mice in contrast were neither affected by age nor by the application of probiotics.^193^ Overall, the effects of gut and oral microbiome dysbiosis are comparable and could be both causally associated to AD pathogenesis in animal model. Understanding how oral microbiome influences the gut flora and vice versa will be instrumental to unravel the causal relationship underlying abnormal hots‐pathogen interactions leading to chronic inflammation and age‐dependent degenerative diseases, as AD.

## 7 PREVENTIVE ASPECTS FROM THE MICROBIOLOGICAL POINT OF VIEW

A proof of concept is targeting the potential causative agent by therapeutic measures, which would mean among others application of antibiotics targeting the causative microorganism. A 3‐month therapy with doxycycline and rifampicin (effective antibiotics against *Chlamydia pneumophila*) in 101 patients resulted in an improvement of cognitive abilities but did not change direct detection of *C pneumophila* or immunoglobulins to that species.^194^ The authors explain it with non‐antimicrobial effects of the antibiotics,^194^ but an influence on the composition of microbial communities can also be discussed. Antibiotics affect gut microbiota up to 180 days after application.^195^ However, in such a concept the global development of antibiotic resistance driven by the use of antibiotics should^196^ not be neglected.

Another possibility is targeting the microbial virulence factors. Many efforts were put on the development of gingipain inhibitors. Initially, it was shown that gingipain inhibitors blocked the cytotoxicity of RgpB and Kgp as well as the cell death of SH‐SY5Y‐cells (a human neuroblastoma cell line) by *P gingivalis*.^117^ Then, 8‐week‐old female BALB/c mice had been orally infected with *P gingivalis* for 6 weeks, after 5 weeks gingipain inhibitors had been applied twice daily for 5 weeks.^117^ The COR388 inhibitor (targeting Kgp, Cortexyme Inc) was shown to be effective, it reduced concentration dependently the levels of *P gingivalis*, Aβ1‐42 and TNFα in the brain.^117^ According to ClinicalTrials.gov ^197^ a clinical phase II/III study (NCTC03823404) with application of COR388 is still running. More than 500 patients with mild to moderate AD are included, and the experimental groups receive the inhibitor twice per day for 48 weeks.^197^ However, according to the published information of the Internet,^198^ the company ended the development of the drug as it failed to provide a cognitive benefit and due to liver toxicity. Here, it may be speculated that the target bacterium might not play such an exclusive role, while on the other hand, it has to be kept in mind that in the oral cavity the microorganisms reside in a well‐organized, complex biofilm consisting of many different microorganisms in close contact to each other, thus limiting drug penetration and efficacy.^199^

Plant‐derived products have been also proposed as alternatives to modulate microbiota and / or host response. A mixture of Traditional Chinese Medicine herbs (“Yuan‐hu Zhi Tong San” [YZT]), having among others also anti‐inflammatory properties, was tested in two transgenic mice models of AD.^200^ YZT mixture was proven to promote clearance of aggregate tau via regulation of the ubiquitin proteasomal system and to improve cognitive functions.^200^ Another Chinese formulation, Qisheng Wan formula decreased deposition of Aβ_1‐42_ in an AD rat model, it ameliorated cognitive damages, and suppressed inflammatory markers. In the gut microbiota, it increased α‐diversity and there was a higher relative abundance of *Firmicutes* and *Bacteroidetes*. Also a substance of interest is curcumin, a polyphenolic compound with anti‐inflammatory and anti‐oxidative properties, which was applied in two concentrations to 6‐month‐old APP/PS1 mice.^201^ The high dose of curcumin improved learning abilities of mice, the low dose decreased amyloid plaques in the hippocampus. The gut microbiota differed between the three groups (high dose of curcumin, low dose of curcumin, control). After curcumin administration, the diversity showed a tendency to increase, the relative abundance of *Bacteroidaceae*, *Prevotellacae* and *Lactobacillacae* decreased and those of *Rikinellaceae* increased.^201^ Metabolites of curcumin were detected in the gut microbiome.^201^

Probiotics were used to modify the gut microbiome and to underline the potential role of the gut microbiome on the development of AD. But they might also be a preventive or therapeutic option. The effect of probiotics was shown to be related to an improvement of glucose uptake in the brain and a hindering of an increase in glycated hemoglobin and advanced glycosylation end products in AD mice.^202^

Application of a probiotic strain (*Brevibacterium breve* A1) prevented cognitive impairment together with an excessive immune response in hippocampus tissue in a mice model where animals were injected intracerebroventricularly with Aβ_25‐35_.^203^ It is of interest to note that *B breve* modified only negligibly the gut microbiota.^203^

Administration of *Bifidobacterium bifidum* BGN4 and *Bifidobacterium longum* BORI in 5xFAD mice reduced hippocampal neuronal death, improved cognitive and memory abilities of the mice, together with a decreased release of several inflammatory biomarkers.^204^ In the microbiome of the 5xFAD mice the genera *Akkermansia*, *Faecalibaculum*, *Eysipelatoclostridium,* and *Candidatus_Stoquefichus* were enriched, and the genera *Parvibacter*, *Incertae_Sedisa,* and *Oscillibacter* were lowered after the probiotics; in control mice the abundance of the genera NK4A214‐group, *Alistepes*, *Lachnoclostridium*, *Desulfovibrio* decreased.^204^ The administration of *B* *bifidum* BGN4 and *B longum* BORI was also proven in cognitive healthy individuals older than 65 years.^205^ At genus level, the relative abundances of *Eubacterium*, *Alisonella*, *Clostridiales,* and *Prevotellaceae* decreased after the 12 weeks intake of the probiotics.^205^ The scores on mental flexibility improved after the 12 weeks in the probiotic but not in the control group.^205^

## 8 CONCLUDING REMARKS

In conclusion, a number of studies have investigated the role of oral microorganisms related to AD. Considering also the reports on the gut microbiome related to AD, a modifying role of the oral microbiota in interaction with the host response is suggested.

An exclusive causal role of *P gingivalis* remains questionable. Mice models show an induction of alterations related to AD, but they do not completely reflect the situation in humans, while data from humans are inconsistent. It needs to be critically remarked that storage conditions of postmortem brain samples are mostly not reported. Our own study supports the potential invasion of oral bacteria or their components into the brain per se but there was no difference between patients with AD and those without.^130^ The very recent premature discontinuation of a clinical trial on a *P* *gingivalis* inhibitor^198^ appears to underline the assumption that the role of this periodontal pathogen might not be crucial in developing AD.

Several factors contribute to the conversion to cognitive impairment. There are several genes identified having a crosstalk between AD and periodontitis, while most upregulated genes are involved in host response such as binding to IgG, complement system, and chemokine synthesis.^206^ Furthermore, an analysis using publicly available genome‐wide association studies on periodontitis and AD failed to reveal any association of genetically predicted AD with the risk of periodontitis.^207^ A genetic susceptibility for developing AD (LOAD) is also known and is underlined by findings from mice models. These mice (eg, APP/PS1 or E4FAD transgenic mice) are able to develop alterations in the brain typical for AD together with cognitive impairment.^187^, ^189^ APOE4 allele is a well‐known risk factor for AD,^16^ but there is no report on an association with periodontal disease. Obviously, more comprehensive studies are needed to shed more light on these issues.

Microbiome analyses of the gut and of the oral microbiome show deviations in cognitive impaired patients in comparison with cognitively healthy individuals. The assumption that the presence of an APOE4 allele may affect the microbiota is supported by findings on a decrease of α‐diversity both in the gut^183^ and the oral microbiome (saliva,^131^ biofilm^133^). However, it has to be kept in mind that neither the findings on the α‐diversity nor those on differences in the composition of the microbiota are consistent. Moreover, the composition of the gut microbiota is strongly dependent on lifestyle factors such as nutrition. Individuals preferring Mediterranean diet have a lower Firmicutes/Bacteroidetes ratio in their gut microbiome, a high intake of animal protein is associated with a higher Firmicutes/ Bacteroides ratio.^208^ In overweighted and obese patients, a dietary treatment with Mediterranean diet reduced counts of *P gingivalis*, *Pr intermedia,* and *T denticola* in saliva.^209^ But it is of interest to note that Mediterranean diet was negatively associated with development of cognitive impairment and dementia in an older population.^210^ This as well as the modification of the gut microbiome resulting in an improvement of cognitive abilities in mice or even in human by application of probiotics may show that a symbiotic microbiota seems to be beneficial in maintaining cognitive health. It can also be anticipated that early diagnosis and therapy of periodontitis is crucial to retard disease progression. Since periodontitis is the result of a disturbed microbial homoeostasis,^163^ an improvement of personal oral hygiene, coupled with professional biofilm and calculus removal followed by an individually tailored supportive therapy are the key parts of successful periodontal therapy.^211^ While mechanical root debridement leads to a microbiome comparable to that of periodontally healthy subjects,^212^ personal oral hygiene and instrumentation decrease inflammatory mediators in the oral cavity.^213^ Dental therapy in mild AD improved periodontal indices and quality of life.^214^ Despite the fact that periodontal therapy might not affect brain aging it may still have a favorable effect on brain atrophy.^215^

In summary, in elderly and, in particular, in patients with cognitive impairment, periodontal therapy is of overwhelming importance for improving their quality of life. An effect of periodontal therapy on the oral microbiome and the host response related to cognitive parameters is paramount to understand causal relationships with age‐dependent morbidities and should be elucidated in longitudinal clinical trials.

## CONFLICT OF INTEREST

The authors declare no potential conflict of interest with respect to the authorship and/or publication of this article.
